# Effects of dexmedetomidine on postoperative sleep quality: a systematic review and meta-analysis of randomized controlled trials

**DOI:** 10.1186/s12871-023-02048-6

**Published:** 2023-03-21

**Authors:** Huizi Liu, Hanwei Wei, Shaojie Qian, Jintao Liu, Weicai Xu, Xiaopan Luo, Junbiao Fang, Qiaoyan Liu, Fang Cai

**Affiliations:** Center for Rehabilitation Medicine, Department of Anesthesiology, Zhejiang Provincial People’s Hospital, Affiliated People’s Hospital, Hangzhou Medical College, Hangzhou, 310014 Zhejiang China

**Keywords:** Dexmedetomidine, Sleep quality, Sleep disturbance, Polysomnography, Meta-analysis

## Abstract

**Study objectives:**

To assess the effect of dexmedetomidine (DEX) on postoperative sleep quality using polysomnography (PSG) to identify possible interventions for postoperative sleep disturbances.

**Methods:**

An electronic search of PubMed/MEDLINE, EMBASE, Cochrane Library and Web of Science was conducted from database inception to November 20, 2022. Randomized controlled trials (RCTs) on the effect of DEX administration on postoperative sleep quality using PSG or its derivatives were included. No language restrictions were applied. The sleep efficiency index (SEI), arousal index (AI), percentages of stage N1, N2 and N3 of non-rapid eye movement (NREM) sleep, and rapid eye movement (REM) sleep were measured in our meta-analysis.

**Results:**

Five studies, involving 381 participants were included. Administration of DEX significantly improved SEI, lowered AI, decreased the duration of stage N1 sleep and increased the duration of stage N2 sleep compared to placebo groups. There were no significant differences in the duration of stage N3 sleep and REM sleep. DEX administration lowered the postoperative Visual Analogue Scale (VAS) score and improved the Ramsay sedation score with no adverse effect on postoperative delirium (POD). However, high heterogeneity was observed in most of the primary and secondary outcomes.

**Conclusions:**

Our study provides support for the perioperative administration of DEX to improve postoperative sleep quality. The optimal dosage and overall effect of DEX on postoperative sleep quality require further investigation using large-scale randomized controlled trials.

**Supplementary Information:**

The online version contains supplementary material available at 10.1186/s12871-023-02048-6.

## Introduction

Sleep disturbances, including sleep deprivation, disruption, and abnormal architecture, are prevalent in postoperative patients [1]. Studies using polysomnography (PSG) have shown that the sleep pattern of patients is characterized by a disorganized circadian rhythm, prolonged sleep latency, fragmented sleep, decreased sleep efficiency, abnormally increased stages 1 and 2 of non-rapid eye movement (NREM) sleep (also called stage N1 and N2 sleep), decreased or absent stage 3 of NREM sleep (also called stage N3 sleep or slow-wave sleep) and rapid eye movement (REM) sleep [2, 3]. Postoperative sleep disturbances can result in significant adverse outcomes, including delirium, cardiovascular events, impaired immune function, prolonged mechanical ventilation, and postoperative physical and mental health decline [4–6]. Therefore, interventions to improve postoperative sleep quality are attracting considerable attention from anesthesiologists.

Dexmedetomidine (DEX), an α-2 adrenergic agonist with high specificity, has been widely used as a sedative, anxiolytic, sympatholytic, and analgesic-sparing agent in clinics [7]. Compared to gamma-aminobutyric acid (GABA) agonists, DEX more closely resembles natural NREM sleep [8, 9]. Several studies have reported favorable effects of DEX on sleep quality in patients after surgery or in the intensive care unit (ICU), as evaluated by objective tools, sleep questionnaires, or subjective assessments [10–14]. However, no meta-analysis of randomized controlled trials (RCTs) has focused on the effect of DEX on sleep quality in postoperative patients, and the optimal dosage and overall effect of DEX remain unclear.

To summarize the available evidence and guide clinical practice, we conducted this systematic review and meta-analysis to evaluate the effects of DEX on postoperative sleep quality using PSG or its derivatives to identify possible interventions for postoperative sleep disturbance. We aimed to provide aggregated data and make a more validated conclusion regarding the administration of DEX to improve postoperative sleep.

## Methods

### Standard protocol approval and registration

This systematic review and meta-analysis followed the Preferred Reporting Items for Systematic Reviews and Meta-analyses (PRISMA) reporting guideline [15]. The protocol was registered in PROSPERO (registration number: CRD42022373253).

### Search strategy

The search was conducted using PubMed/MEDLINE, EMBASE, Cochrane Library and Web of Science from inception to November 20, 2022. The full search strings for each database are available in Appendix S1. Medical Subject Headings (MeSH) terms and corresponding keywords were combined to find potentially available articles. For instance, researches about DEX were searched using “dexmedetomidine” (MeSH term) OR “MPV-1440” OR “MPV 1440” OR “MPV1440” OR “Precedex” OR “Dexmedetomidine Hydrochloride” OR “Hydrochloride Dexmedetomidine”. All terms were explored in the “Title/Abstract” or “Keywords” sections. Moreover, references from identified studies and relevant published reports were manually searched to identify possibly eligible trials in the present topic.

### Study selection

Studies were included in our meta-analysis according to the following criteria: (1) participants: adult patients who underwent elective surgery; (2) intervention: pre-, peri-, or postoperative administration of DEX; (3) comparison: placebo (normal saline), other sedative drugs or analgesics; (4) outcome: sleep quality should be objectively evaluated through PSG or its derivatives, including at least one of the following parameters: sleep efficiency index (SEI) or total sleep time (TST), arousal index (AI), the percentages of stage N1, N2, and N3 of NREM sleep and REM sleep; (5) study design: patients should be randomly allocated to different treatments or different sequences of treatments; (6) studies should be published or accepted for publication in a peer-reviewed journal; (7) studies should be theses or dissertations with full-text access. We made no restrictions on sample size, treatment duration, or publication date because of the anticipated small number of studies that used PSG data. We also made no geographical or cultural restrictions because we were interested in a global perspective on postoperative sleep quality and its treatments.

Duplicate articles were removed, and the titles and abstracts of potentially eligible articles were independently screened by two reviewers (Huizi Liu and Hanwei Wei). The full-text articles from the remaining studies were retrieved and reviewed. Only studies that fulfilled the inclusion criteria were selected for our systematic review and meta-analysis. Any discrepancies during article selection were reassessed by another author (Fang Cai) and resolved through discussion to reach a consensus.

### Data extraction and risk of bias assessment

Data for assessing the outcomes were independently extracted and recorded by two reviewers (Huizi Liu and Hanwei Wei) from the included studies. Any discrepancies were reassessed by another author (Fang Cai) and resolved through discussion and consensus. The following information of each selected article was collected: first author; year of publication; study design; geographical location; sample size; participant characteristics, including mean age, gender distribution, American Society of Anesthesiologists (ASA) classification, type of surgery; inclusion and exclusion criteria; intervention strategies (type, dosage, approach, frequency and duration); primary and secondary outcomes; sleep evaluation tool; results data and statistical data.

The following outcomes were used to evaluate sleep quality: (1) SEI or TST: SEI was calculated as the ratio of TST/total recording time; (2) AI was defined as the average number of arousals per hour of sleep; and (3) the percentages of stage N1, N2, and N3 of NREM sleep and REM sleep. The primary outcome should include at least one of these parameters. The secondary outcomes included postoperative Ramsay sedation scores, Visual Analog Scale (VAS) scores, postoperative delirium (POD), and postoperative nausea and vomiting (PONV). We attempted to contact the authors by e-mail if there were missing data. If a study did not report standard deviations (SDs), we imputed the SDs of the included trial comparing the same treatments.

Two reviewers (Huizi Liu and Hanwei Wei) independently evaluated the methodological quality of the included studies using the Cochrane risk-of-bias tool. The risk of bias for each RCT covered seven domains: random sequence generation, allocation concealment, blinding, outcome assessment, incomplete outcome data, selective reporting and other biases. The risk of bias for each item was rated as “high,” “low” or “unclear”. Discrepancies were resolved through consensus.

### Data synthesis and analysis

Data synthesis and statistical analyses were performed using the Review Manager (RevMan, version 5.4) software (Cochrane Library, Oxford, UK). The data extracted from the literature in the present review were continuous variables; therefore, we calculated the mean differences (MDs) and 95% confidence intervals (CIs). If the primary outcomes were reported as median (interquartile range, IQR), the methodology of Wan et al. [16] and Luo et al. [17] was used to convert the median (IQR) to mean ± SD. Statistical differences were not considered significant if the 95% CI included zero for the MD. Forest plots were used to present the pooled results and corresponding 95% CIs. Cochrane Q test (*p* < 0.10 for a statistical significance) and *I*² test were conducted to evaluate the heterogeneity among included research. As described in the Cochrane review guidelines, *I*² > 50% indicated a significantly high heterogeneity, and the corresponding outcome was analyzed with a random effects model, whereas the fixed effects model was applied. In addition, an egger test and begg test were performed to explore possible publication bias. A trial sequential analysis of PSG parameter was performed to determine whether the sample size was adequate and the results were stable.

Subgroup analysis was performed according to the severity of illness, administration regimen of DEX, type of surgery, ward environment, etc. Heterogeneity was resolved by subgroup analysis when two or more studies were included in each subgroup. Additionally, based on the results of the quality evaluation, a sensitivity analysis was performed by excluding articles with a significantly high risk of bias.

## Results

### Study selection and characteristics

**Fig. 1:**
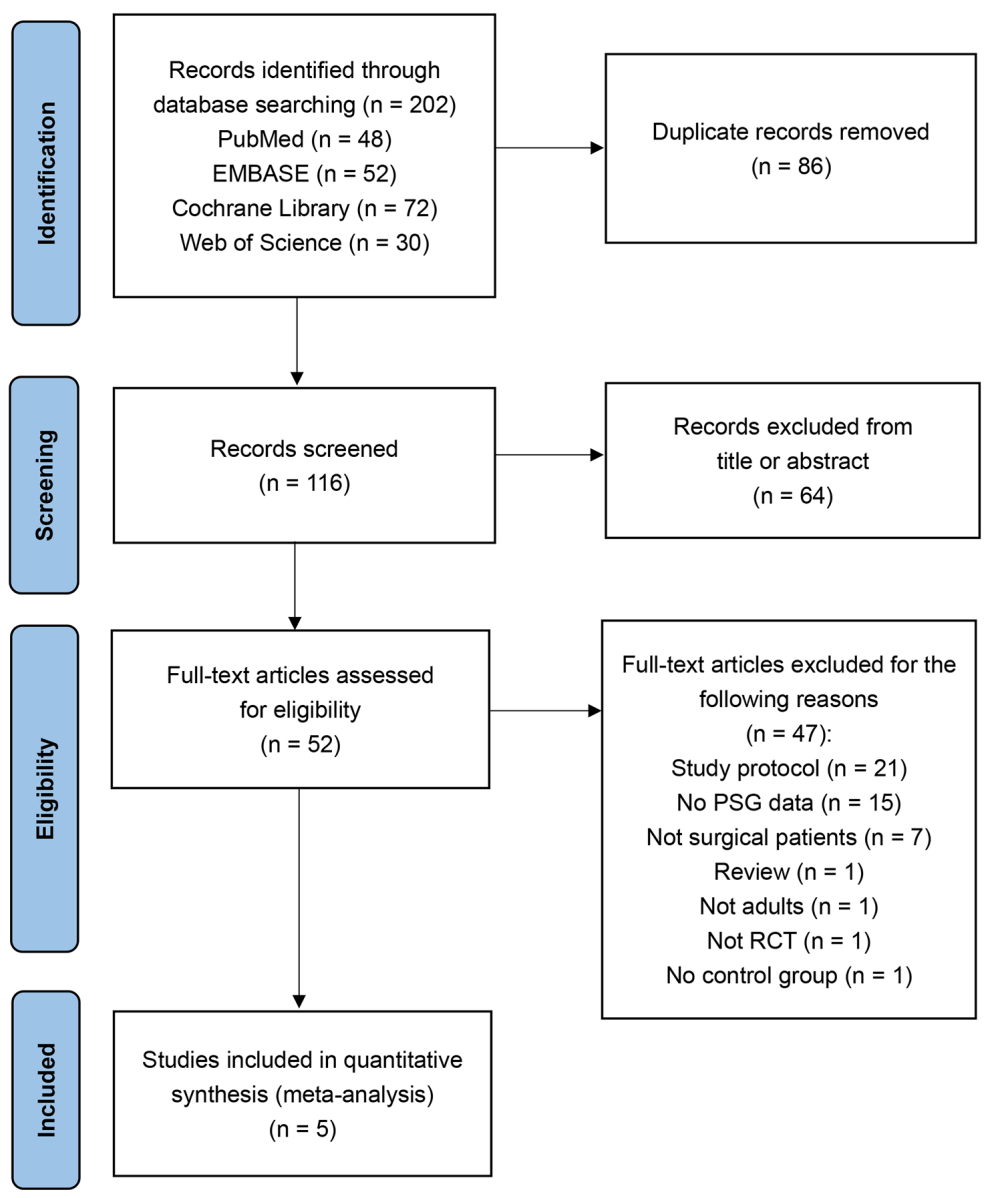
The flow diagram of identifying studies through systemic search in multiple databases

A total of 202 potentially relevant articles were identified initially from the electronic databases and 116 articles remained after removing duplicates. 64 articles were excluded after reading titles and abstracts because they did not meet the inclusion criteria. After reviewing the full text of the remaining 52 potentially eligible articles, 21 were excluded because they were study protocols, one was a review, 15 had no PSG data, seven were not for surgical patients, one was not an adult study, one was not an RCT, and one had no control group. Five eligible studies were identified [13, 14, 18–20]. The selection process is illustrated in Fig. 1.

The relevant baseline characteristics of each study, including descriptions of the DEX administration regimens, are summarized in Table 1. After excluding the number of dropouts reported in each study, the meta-analysis covered a sample size of 381 patients and included five RCTs. Two studies used DEX postoperatively, two used it as an adjuvant in patient controlled analgesia (PCA), and one study used DEX intraoperatively. All the PSG parameters were collected on the first night after surgery. The VAS score and Ramsay sedation score were assessed at 6 h after surgery. For PSG parameters, five studies reported SEI, AI, and the percentage of REM sleep; four studies reported the percentages of stage N1, N2, and N3 of NREM sleep, and two studies reported TST. Postoperative analgesia was provided with a patient-controlled intravenous or epidural analgesia pump to maintain the VAS ≤ 4 at rest or behavior pain score ≤ 6, except for patients in one study undergoing endoscopic sinus surgery. Supplemental analgesics were administered for patients when necessary.

**Table 1:** Baseline characteristics of included trials

First author, year of publication	Country	Type of surgery	Age range(yr)	Males(%)	Control group	No. randomized	No. in DEX group	No. in control group	ICU stay	DEX starting time	DEX dosage	Variables provided
Wu XH^[[20]]^, 2016	China	Non-cardiac Surgery	≥ 65	57.9	Placebo	61	31	30	Yes	Postoperative	0.1 µg/kg/h, lasting 15 h	SEI, TST, AI, N1, N2, N3, REM
Chen ZC^[[13]]^, 2017	China	Abdominal hysterectomy	30–55	NR	Placebo	59	30	29	No	PCA	Continuous dose: 0.05 µg/kg/h Bolus dose: 0.05 µg/kg	SEI, AI, N1, N2, N3, REM, Ramsay, VAS
Jiang ZM^[[18]]^, 2018	China	Abdominal surgery	60–70	58.8	Placebo	97	Low dose group: 32 High dose group: 33	32	No	PCA	Low dose group: 0.072 µg/kg/h continuous dose, 0.024 µg/kg bolus dose High dose group: 0.144 µg/kg/h continuous dose, 0.048 µg/kg bolus dose	SEI, AI, N1, N2, N3, REM, VAS
Sun YM^[[19]]^, 2022	China	Non-cardiac surgery	≥ 18	51.4	Placebo	68	33	35	Yes	Postoperative	0.1–0.2 µg/kg/h, lasting 72 h	SEI, TST, AI, N1, N2, N3, REM
Wu Y^[[14]]^, 2022	China	Endoscopic sinus surgery	18–65	55	Placebo	96	48	48	No	Intraoperative	Loading dose: 0.5 µg/kg, continuous infusion: 0.2 µg/kg/h	SEI, AI, REM, Ramsay, VAS, PONV, POD

NR, not reported; PCA, patient controlled analgesia; SEI, sleep efficiency index; TST, total sleep time; AI, arousal index; N1, stage 1 of non-rapid eye movement sleep; N2, stage 2 of non-rapid eye movement sleep; N3, stage 3 of non-rapid eye movement sleep; REM, rapid eye movement sleep; VAS, visual analogue scale; PONV, postoperative nausea and vomiting; POD, postoperative delirium

### Meta-analysis results

**Fig. 2:**
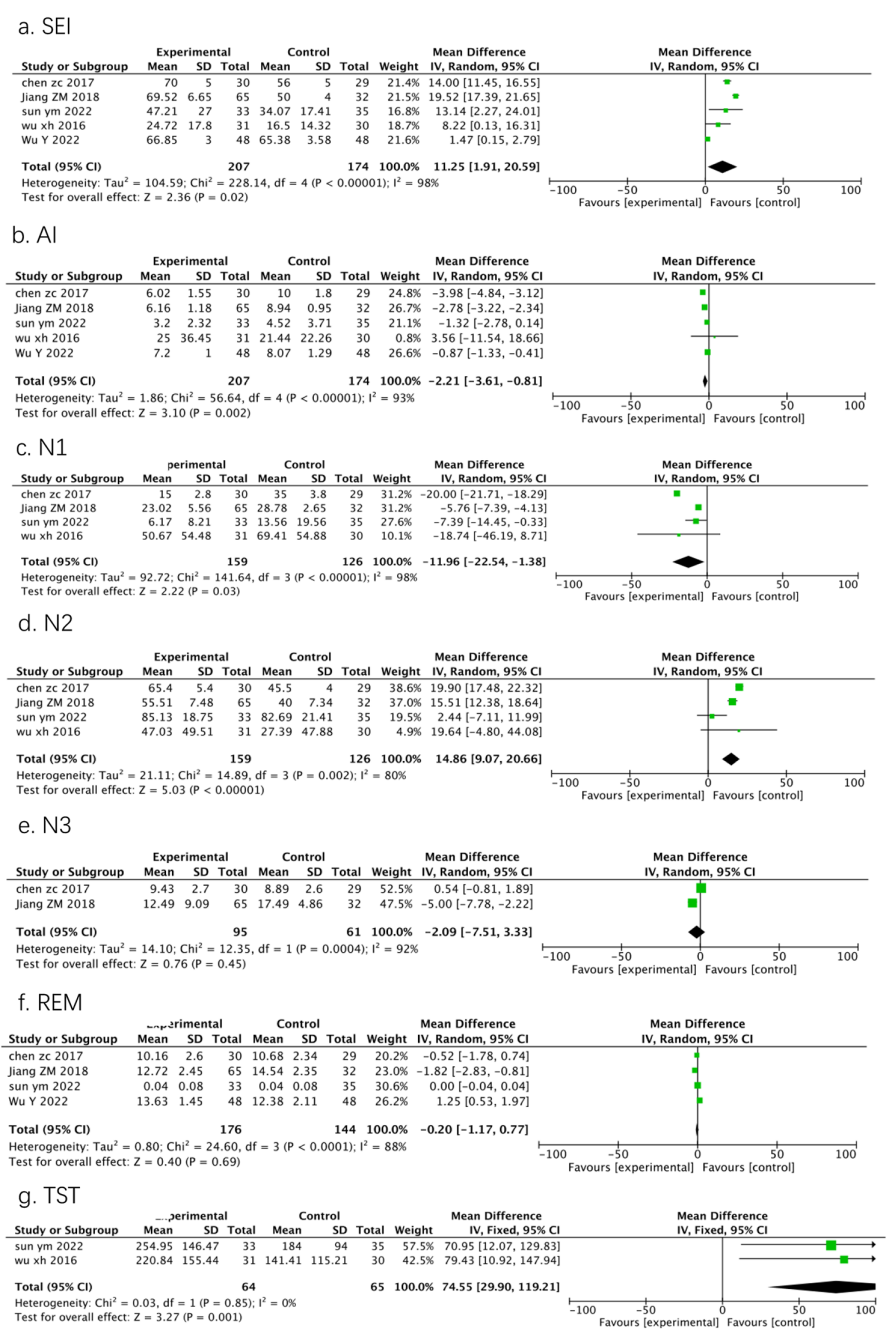
Forest plots for effects of DEX compared with placebo on sleep quality. (a) SEI, sleep efficiency index; (b) AI, arousal index; (c) N1, stage 1 of non-rapid eye movement sleep; (d) N2, stage 2 of non-rapid eye movement sleep; (e) N3, stage 3 of non-rapid eye movement sleep; (f) REM, rapid eye movement sleep; (g) TST, total sleep time; CI, confidence interval

**Fig. 3:**
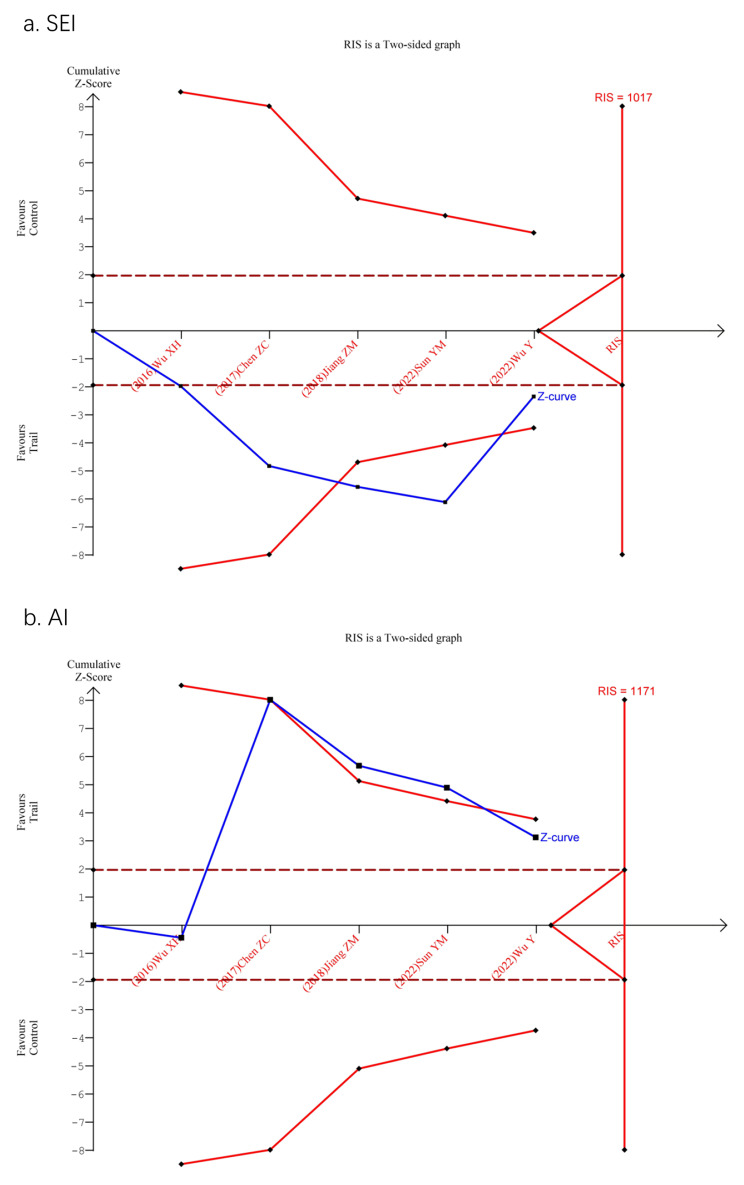
Trial sequential analysis for SEI and AI. (a) SEI, sleep efficiency index; (b) AI, arousal index

Five studies reported the effect of DEX on SEI and AI compared to placebo groups, and the forest plots are presented in Fig. 2a, b. Administration of DEX significantly improved SEI (11.25%, 95%CI = 1.91–20.59, *p* = 0.02) and lowered AI (-2.21, 95%CI = -3.61- -0.81, *p* = 0.002). Four studies reported the effect of DEX on the percentages of stage N1, N2, and N3 of NREM sleep compared to placebo groups. The comparison showed that the duration of stage N1 sleep was shortened (-11.96%, 95%CI = -22.54- -1.38, *p* = 0.03) and the duration of stage N2 sleep was longer (14.86%, 95%CI = 9.07–20.66, *p* < 0.00001) in the DEX group than in the placebo group (Fig. 2c, d**)**. There were no significant differences in the duration of stage N3 sleep (-2.09%, 95%CI = -7.51-3.33, *p* = 0.45) and REM sleep (-0.20%, 95%CI = -1.17-0.77, *p* = 0.69), as shown in Fig. 2e, f. Two studies reporting TST showed that the administration of DEX significantly prolonged TST (74.55 min, 95%CI = 29.90-119.21, *p* = 0.001) (Fig. 2g). The trial sequential analysis was performed on the SEI and AI (Fig. 3). The cumulative Z-curve crossed both the conventional meta-analysis boundary and the trial sequential monitoring boundary before the accumulated information reached the required information size, which demonstrated that our results were considered to be stable.

**Fig. 4:**
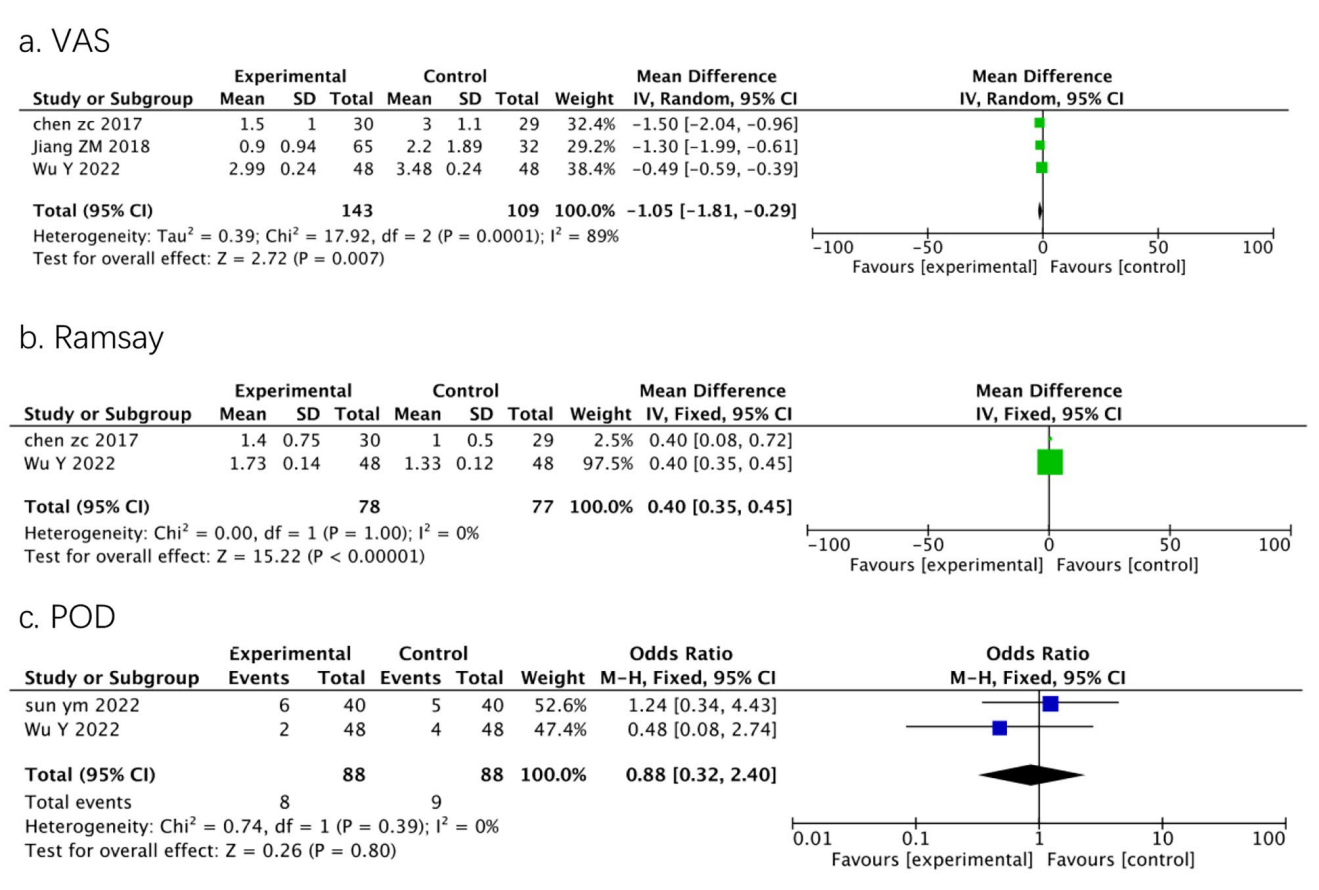
Forest plots for effects of DEX compared with placebo on secondary outcomes. a. VAS score, Visual Analog Scale score; c. POD, postoperative delirium, CI, confidence interval

For secondary outcomes, four studies collected postoperative VAS scores, two reported Ramsay sedation scores and the incidence of POD, and one mentioned the incidence of PONV. Our meta-analysis demonstrated that DEX administration lowered the postoperative VAS score (-1.05, 95%CI = -1.81- -0.29, *p* = 0.007) and improved the Ramsay sedation score (0.40, 95%CI = 0.35-0.45, *p* < 0.00001) with no adverse effect on POD (odds ratio = 0.88, 95%CI = 0.32-2.40, *p* = 0.80), as shown in Fig. 4. The meta-analysis for PONV was not conducted because of insufficient data. However, considering the high heterogeneity of most PSG parameters to evaluate sleep quality and secondary outcomes, we indicated that one or more studies may have influenced the results; thus, subgroup analysis was conducted to interpret the heterogeneity.

### Subgroup analysis

Table 2 presents subgroup analyses of postoperative PSG data stratified by severity of illness. Patients in two studies were admitted to the ICU, and others in three studies returned to the ward. We observed statistically different results on SEI (9.98%, 95%CI = 3.48-16.47, *p* = 0.003) and duration of stage N1 sleep (-8.09%, 95%CI = -14.93- -1.26, *p* = 0.02) in the ICU subgroup, with a dramatically decreased heterogeneity (*I*^2^ = 0). There were statistical differences on AI (-2.51, 95%CI = -4.17- -0.86, *p* = 0.003) and duration of stage N2 sleep (17.82%, 95%CI = 13.53-22.12, *p* < 0.00001) in the non-ICU subgroup, but the heterogeneity remained high. The heterogeneity on AI and duration of stage N2 sleep was decreased in the ICU subgroup, however, no significant effect was observed in these two parameters. In addition, no statistical difference was found in the duration of stage N3 and REM sleep. Given that the stage N3 and REM sleep were barely achieved in the ICU patients, several parameters were not estimable or applicable, as shown in Table 2. From the results above, we cannot come up with the conclusion that the severity of illness is a source of heterogeneity in our meta-analysis. However, the overall effect of DEX on postoperative sleep quality seems to be beneficial.

**Table 2:** Subgroup analysis stratified by severity of illness

	Subgroup	Sample size		Weight (%)	MD with [95%CI]	Z	*I*^2^(%)	*P* value
		**DEX**	**Control**					
SEI	ICU	64	65	35.5	9.98 [3.48, 16.47]	3.01	0	0.003
	Non-ICU	143	109	64.5	11.64 [-0.39, 23.66]	1.90	98	0.06
AI	ICU	64	65	21.9	-1.27 [-2.73, 0.18]	1.72	0	0.09
	Non-ICU	143	109	78.1	-2.51 [-4.17, -0.86]	2.98	96	0.003
N1	ICU	64	65	37.6	-8.09 [-14.93, -1.26]	2.32	0	0.02
	Non-ICU	95	61	62.4	-12.88 [-26.83, 1.08]	1.81	99	0.07
N2	ICU	64	65	24.4	7.21 [-7.88, 22.30]	0.94	39	0.35
	Non-ICU	95	61	75.6	17.82 [13.53, 22.12]	0.13	79	< 0.00001
N3	ICU	64	65	0	NE	NA	NA	NA
	Non-ICU	95	61	100	-2.09 [-7.51, 3.33]	0.76	92	0.45
REM	ICU	33	35	30.6	0 [-0.04, 0.04]	0	NA	1
	Non-ICU	143	109	69.4	-0.34 [-2.32, 1.64]	0.33	92	0.74

SEI, sleep efficiency index; AI, arousal index; N1, stage 1 of non-rapid eye movement sleep; N2, stage 2 of non-rapid eye movement sleep; N3, stage 3 of non-rapid eye movement sleep; REM, rapid eye movement sleep; MD, mean difference; CI, confidence interval; NE, not estimable; NA, not applicable

### Sensitivity analysis

**Fig. 5:**
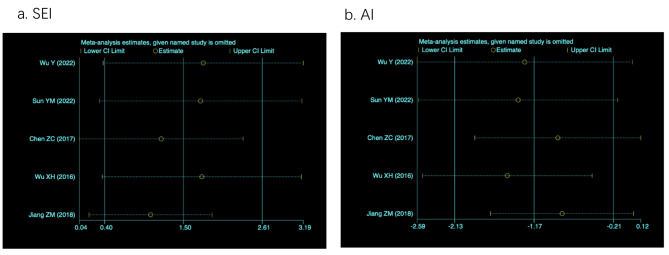
Sensitivity analysis for SEI and AI. a. SEI, sleep efficiency index; b, AI, arousal index; CI, confidence interval

Sensitivity analysis for the PSG parameter was also conducted. We did not find any single study that could make a significant impact on the primary outcome, indicating that our results were reliable and statistically stable, as shown in Fig. 5.

### Risk of bias

**Fig. 6:**
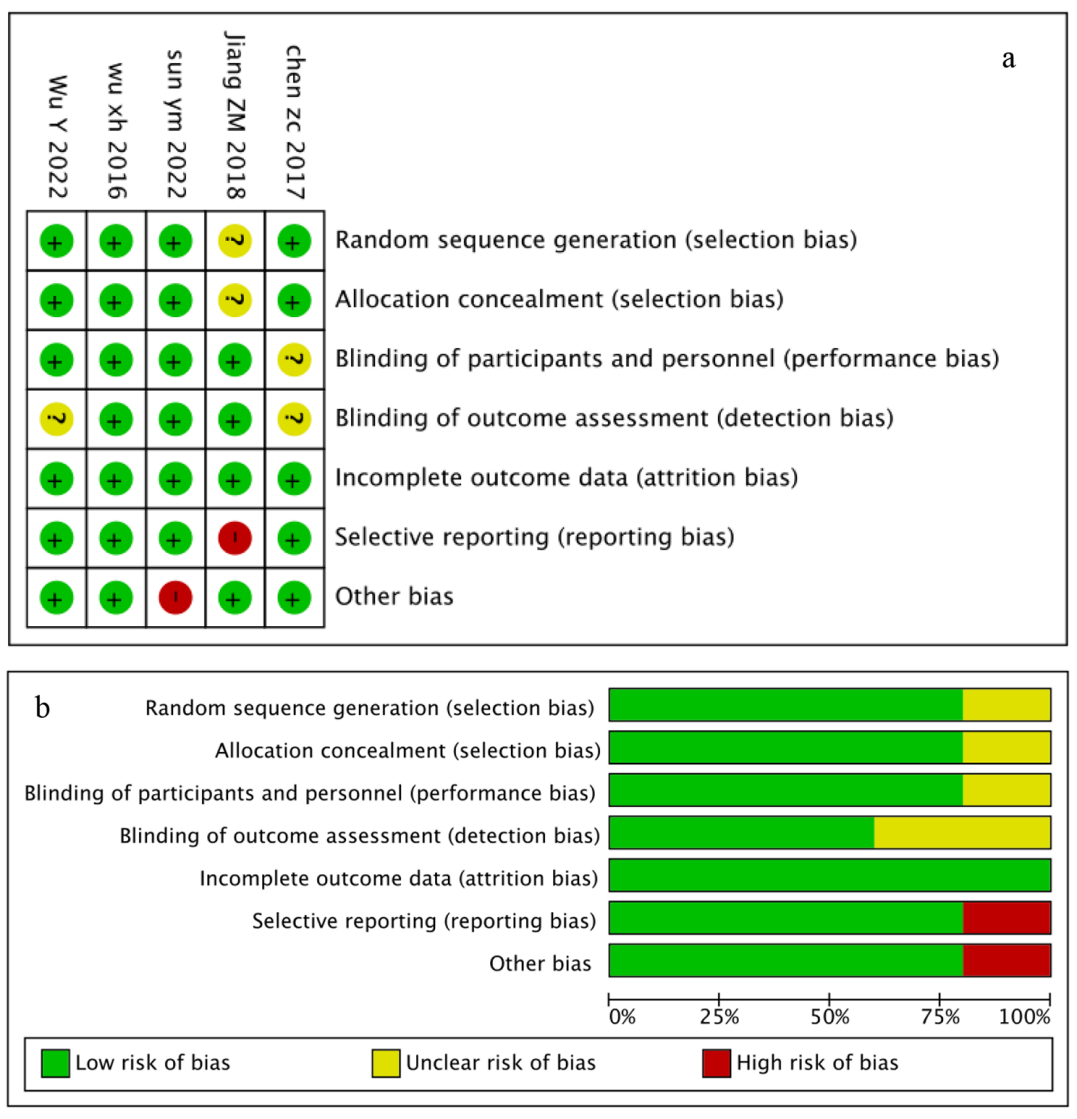
The risk of bias in the included studies. a: risk of bias for each study; b: risk of bias summary

The risk of bias is summarized in Fig. 6, which has been described for individual studies, and a summary, respectively. All trials were randomized, and most (4 out of 5, 80%) reported the methods of randomization. Four trials reported allocation concealment. Participants and personnel were blinded in four trials. Three trials were blinded to the outcome assessment. The remaining trials contained several domains that lacked clarity. The risk of bias for selective reporting was rated high in one study because of missing data in the control group. Other bias was rated high in one study because several patients were excluded for premature drug interruption, death within 30 days, and refusion to the follow-up test. Egger tests were performed and showed no publication bias based on SEI (*p* = 0.052) and AI (*p* = 0.246). Begg tests also demonstrated no publication bias on SEI (*p* = 0.221) and AI (*p* = 0.806).

## Discussion

To the best of our knowledge, this is the first meta-analysis to evaluate the effects of DEX on postoperative sleep quality based on PSG data. The results of our systematic review and meta-analysis of RCTs showed that perioperative administration of DEX may be adequate to improve postoperative sleep quality within a short period by increasing the percentage of stage N2 sleep and sleep efficiency, prolonging the duration of TST, and decreasing the percentage of stage N1 sleep and sleep arousals. Additionally, the administration of DEX could provide effective postoperative analgesia by reducing the VAS score.

Many variables can be used to measure sleep quality. PSG can assess sleep architecture objectively and accurately reflect the percentage of each sleep stage, which is more helpful for our subsequent meta-analysis. In addition to PSG, objective tools include portable sleep monitor and the bispectral index (BIS). A portable sleep monitor can evaluate sleep efficiency, percentage of REM sleep, and unstable and stable sleep, whereas the BIS measures the level of sedation, sleep depth, SEI, and TST. Subjective tools are mostly questionnaires and scales, including the Pittsburgh Sleep Quality Index (PSQI), Richards Campbell Sleep Questionnaire (RCSQ), St. Mary Hospital Sleep Questionnaire (SMH), numeric rating scale (NRS), insomnia severity index (ISI), Athens insomnia scale (AIS), consensus sleep diary (CSD), and other simple questions. Apart from our results using PSG, other questionnaires of the above-mentioned tools also showed positive effects of DEX on postoperative sleep quality [21–28].

A normal sleep pattern and cycle are crucial to maintain normal physiological and mental functions. Stage N1 is regarded as light sleep (drowsiness) and stages N2 and N3 represent deeper sleep. Slow-wave sleep (SWS) is considered to be the deepest sleep stage. Dreaming usually occurs in REM sleep [29]. Previous studies have reported that patients experienced severe sleep disturbances with a pronounced decrease in REM sleep immediately after surgery and a tendency for a rebound phenomenon within the first week after surgery [30], even in some fast-track surgeries involving regional anesthesia, opioid-sparing multimodal analgesia, mobilization on the day of surgery, and a planned length of stay of 1–3 days [31]. At the same time, stage N1 sleep was significantly longer [1, 13]. Electroencephalogram (EEG) patterns associated with DEX-induced sleep include spindle and slow-delta oscillations, which are similar to physiological stage N2 sleep [32]. DEX binds to receptors in the locus coeruleus and inhibits norepinephrine release, which causes GABA output from the ventrolateral preoptic nucleus, resulting in NREM sleep patterns [10]. Norepinephrine plays a permissive role during REM sleep [33]; therefore, inhibition of its release by DEX may make REM sleep difficult to achieve. These results are consistent with our meta-analysis findings that DEX provides a slightly deeper and more physiological sleep pattern and improves postoperative sleep architecture by shortening stage N1 sleep and prolonging stage N2 sleep with no significant effect on REM sleep. Additionally, increased stage N2 sleep and decreased REM sleep were found in healthy volunteers using an oral dosage formulation of DEX [34]. Stage N3 sleep is promoted by DEX in a dose-dependent manner [35, 36], which may be beneficial for cognitive function and synaptic plasticity [37].

The dosage and timing of administration were related to the effects of DEX on postoperative sleep quality. A real-world cohort study covering 7418 patients undergoing non-cardiac major surgery demonstrated that the incidence of severe sleep disturbance in the low-dose (0.2–0.4 µg/kg/h) DEX group was significantly lower than that in the medium- (0.4–0.6 µg/kg/h) and high-dose (0.6–0.8 µg/kg/h) DEX groups [26]. Jiang et al. [18] compared the effects of oxycodone in combination with different doses of DEX on postoperative sleep quality, and the results indicated that larger doses of DEX did not further improve sleep but increased the risk of hypotension. From these results, it can be concluded that low-dose DEX may be the optimal treatment for postoperative sleep disturbance. Song et al. [28] found that intraoperative use of DEX during the daytime (8:00–12:00) operation could improve sleep efficiency and subjective sleep quality and promote the analgesic property of sufentanil-based PCA than that during the nighttime (18:00–22:00) operation under general anesthesia, which might be explained by the pharmacologic sensitivity influenced by chronobiology and time-dependent variations in pain [38]. However, Tan et al. [39] reported worse sleep on the night of surgery using DEX under spinal anesthesia than with midazolam. A possible explanation is that the natural sleep cycle of patients is disturbed during the daytime with the deeper sedative state provided by DEX. Similar results were obtained in the ICU [10]. Given the above, further studies should focus on the optimal dosage of DEX and the timing of administration to better treat postoperative sleep disturbances.

Postoperative sleep disturbances can lead to hyperalgesia [40]. Therefore, effective postoperative analgesia may positively affect sleep quality. As widely used analgesics, opioids have been reported to negatively impact sleep architecture by decreasing REM and stable sleep, resulting in deteriorated sleep quality in post-surgical patients [1]. The result of our meta-analysis on postoperative analgesia is also consistent with previous studies [28, 41, 42]. DEX, used as an adjuvant for pain management, can contribute to the recovery-promoting effect and treat postoperative sleep disturbances induced by pain.

Critically ill patients exhibit disorganized and poor sleep quality, as evidenced by the lack of sequential progression through sleep stages and low percentages of SWS and REM sleep [5, 6, 43]. Even after discharge, patients report sleep disturbances and continue to experience poor sleep quality [44]. Oto et al. [10] first performed PSG to assess sleep with DEX sedation in mechanically ventilated patients and concluded that night-time infusion of DEX preserved the day-night cycle of sleep but induced severely disturbed sleep architecture without evidence of SWS and REM sleep. However, the absence of a control group and poor control of sedation depth complicates the interpretation of these results. Subsequently, a pilot study [45] demonstrated that night-time DEX administration to achieve the recommended light sedation in critically ill patients increased sleep efficiency and improved sleep quality by reducing sleep fragmentation and shifting sleep from stage N1 to stage N2. DEX modified the 24-h sleep pattern by shifting sleep mainly to the night, partly restoring the normal circadian rhythm. In addition, this study found that DEX sedation did not increase most restorative sleep stages (SWS and REM), which is in line with our meta-analysis. As shown in Fig. 4, two studies involving ICU patients reported longer TST but very low percentages or even the absence of stage N3 and REM sleep. A possible explanation is that the sleep quality of critically ill patients remains low even during sedation with this agent. This result suggests that although the TST may be normal or even increased, critically ill patients are considered to have qualitatively disrupted sleep. Recent studies have reported positive effects of DEX on sleep quality in critically ill patients with or without surgical procedures [12, 46]. Future studies should focus on the duration or frequency of DEX administration to improve the sleep quality in these patients.

The side effects of the perioperative administration of DEX remain controversial. Some studies have suggested that DEX could cause hypotension or bradycardia [13, 18, 20, 47], while others have reported similar respiratory and hemodynamic safety of DEX compared to placebo or other sedatives [23, 39, 42]. Of the included studies in our meta-analysis, two studies reported that the use of DEX slightly increased the occurrence of hypotension and bradycardia without the requirement of intervention [13, 18], while the other studies showed no significant differences with regard to those side effects or the percentage of drug interruption because of the above side effects. Although we did not conclude that DEX had significant side effects or unsafe outcomes, dosage and infusion rate should be considered, since hypotension and bradycardia caused by DEX are common in clinical settings, especially in aged population.

### Limitations

The present meta-analysis had several limitations. First, considering the unified evaluation of sleep quality, only studies using PSG were included, which led to the small sample size of our meta-analysis. The limited number of studies for the majority of outcomes precluded effective comparisons, exploration of heterogeneity, and assessment of small-study effects. Second, DEX regimens and timing of administration, patient population, the severity of illness, type of surgery, and follow-up varied widely across the included trials, which explains the high heterogeneity of most parameters in our results. Furthermore, in our assessment of the risk of bias, some trials had high risk in at least one domain. Due to the lack of detailed reporting, the risk of bias was rated as unclear in several domains. Last but not least, the inclusion and exclusion criteria were strictly observed by all our reviewers, however, the final included studies were all from China. We have done the egger test and begg test, which showed no publication bias.

## Conclusion

In conclusion, our systematic review and meta-analysis provided support for the perioperative administration of DEX in improving postoperative sleep quality by increasing the percentage of stage N2 sleep and sleep efficiency, prolonging the duration of TST, and decreasing the percentage of stage N1 sleep and sleep arousals. In addition, DEX, used as an adjuvant for pain management, provided effective postoperative analgesia. Based on these results, future studies are needed to determine the optimal dosage and regimen, timing of administration, overall efficacy, and safety of DEX in a broad range of patient populations.

## Electronic supplementary material

Below is the link to the electronic supplementary material.

Supplementary Material 1

Supplementary Material 2

## Data Availability

The datasets generated and analysed during the current study are available from the corresponding author on reasonable request.
