# Correction: Effects of dexmedetomidine on postoperative sleep quality: a systematic review and meta-analysis of randomized controlled trials

**DOI:** 10.1186/s12871-023-02070-8

**Published:** 2023-04-03

**Authors:** Huizi Liu, Hanwei Wei, Shaojie Qian, Jintao Liu, Weicai Xu, Xiaopan Luo, Junbiao Fang, Qiaoyan Liu, Fang Cai

**Affiliations:** Center for Rehabilitation Medicine, Department of Anesthesiology, Zhejiang Provincial People’s Hospital, Affiliated People’s Hospital, Hangzhou Medical College, Hangzhou, 310014 Zhejiang China

**Correction: BMC Anesthesiol 23, 88 (2023)**

**https://doi.org/10.1186/s12871-023-02048-6**

Following publication of the original article [1], the authors reported an error under the Discussion section, page 11. Figure 4 citation should be corrected to Figure 2.

The original article [1] has been updated.
