# Supplementary material for: Effects of dexmedetomidine on postoperative sleep quality: a systematic review and meta-analysis of randomized controlled trials
Source: BMC Anesthesiol. 2023 Mar 21;23:88. doi: 10.1186/s12871-023-02048-6 (PMC10029163; doi:10.1186/s12871-023-02048-6)
Supplement: Supplementary file 1 — Supplementary Material 1 [file 12871_2023_2048_MOESM1_ESM.docx]

Appendix S1. Search strategy

Database: PubMed/MEDLINE

Search filter

#1 Search: ((((((Dexmedetomidine[MeSH]) OR (MPV-1440[Title/Abstract])) OR (MPV 1440[Title/Abstract])) OR (MPV1440[Title/Abstract])) OR (Precedex[Title/Abstract])) OR (Dexmedetomidine Hydrochloride[Title/Abstract])) OR (Hydrochloride, Dexmedetomidine[Title/Abstract])

#2 Search: (((Sleep quality[MeSH]) OR (Qualities, Sleep[Title/Abstract])) OR (Quality, Sleep[Title/Abstract])) OR (Sleep Qualities[Title/Abstract])

#3 Search: (((((((randomized controlled trial[Publication Type]) OR (controlled clinical trial[Title/Abstract])) OR (randomized[Title/Abstract])) OR (controlled[Title/Abstract])) OR (trial[Title/Abstract])) OR (placebo[Title/Abstract])) OR (randomly[Title/Abstract])) OR (groups[Title/Abstract])

#1 And #2 And #3

Database: Embase

Search filter

#1 'dexmedetomidine'/exp

#2 'mpv-1440':ti,ab

#3 'mpv 1440':ti,ab

#4 'mpv1440':ti,ab

#5 'precedex':ti,ab

#6 'dexmedetomidine hydrochloride':ti,ab

#7 'hydrochloride, dexmedetomidine':ti,ab

#8 #1 OR #2 OR #3 OR #4 OR #5 OR #6 OR #7

#9 'sleep quality'/exp

#10 'qualities, sleep':ti,ab

#11 'quality, sleep':ti,ab

#12 'sleep qualities':ti,ab

#13 #9 OR #10 OR #11 OR #12

#14 'randomized controlled trial'/exp

#15 #8 AND #13 AND #14

Database: Cochrane library

Search filter

#1 MeSH descriptor: [Dexmedetomidine] explode all trees

#2 (Dexmedetomidine Hydrochloride):ti,ab,kw

#3 (Hydrochloride, Dexmedetomidine):ti,ab,kw

#4 (Precedex):ti,ab,kw

#5 (MPV-1440):ti,ab,kw

#6 (MPV 1440):ti,ab,kw

#7 (MPV1440):ti,ab,kw

#8 #1 OR #2 OR #3 OR #4 OR #5 OR #6 OR #7

#9 MeSH descriptor: [Sleep Quality] explode all trees

#10 (Sleep Qualities):ti,ab,kw

#11 (Qualities, Sleep):ti,ab,kw

#12 (Qualitie, Sleep):ti,ab,kw

#13 #9 OR #10 OR #11 OR #12

#14 #8 AND #13

Database: Web of science

Search filter

#1 TI=(Dexmedetomidine OR MPV-1440 OR MPV 1440 OR MPV1440 OR Precedex OR Dexmedetomidine Hydrochloride OR Hydrochloride, Dexmedetomidine)) OR AB=(Dexmedetomidine OR MPV-1440 OR MPV 1440 OR MPV1440 OR Precedex OR Dexmedetomidine Hydrochloride OR Hydrochloride, Dexmedetomidine

#2 TI=(Sleep quality OR Qualities, Sleep OR Quality, Sleep OR Sleep Qualities)) OR AB=(Sleep quality OR Qualities, Sleep OR Quality, Sleep OR Sleep Qualities

#3 #1 AND #2 (Filter: clinical trial)
