# Supplementary material for: Effects of dexmedetomidine on postoperative sleep quality: a systematic review and meta-analysis of randomized controlled trials
Source: BMC Anesthesiol. 2023 Mar 21;23:88. doi: 10.1186/s12871-023-02048-6 (PMC10029163; doi:10.1186/s12871-023-02048-6)
Supplement: Supplementary file 2 — Supplementary Material 2 [file 12871_2023_2048_MOESM2_ESM.docx]

Selection process

- **Records identified through database searching (n = 202)**
- **Duplicate records removed (n = 86)**
- **Records screened (n = 116)**

**Records excluded after reading title or abstract (n = 64)**

1. Cortinez, L.I., et al., Dexmedetomidine pharmacodynamics: part II: crossover comparison of the analgesic effect of dexmedetomidine and remifentanil in healthy volunteers. Anesthesiology, 2004. 101(5): p. 1077Γאנ1083.

2. Euctr, N.O., Dexmedetomidine versus midazolam til sedasjon av pediatriske intensivpasienter ved Oslo Universitetssykehus. - DEX-studie. https://trialsearch.who.int/Trial2.aspx?TrialID=EUCTR2009-011112-37-NO, 2009.

3. Saadawy, I., et al., Effect of dexmedetomidine on the characteristics of bupivacaine in a caudal block in pediatrics. ACTA ANAESTHESIOLOGICA SCANDINAVICA, 2009. 53(2): p. 251-256.

4. Euctr, A.T., The effect and safety of dexmedetomidine as an additive to ropivacaine for interscalene brachial plexus blocks (regional anesthesia) for shoulder surgery. https://trialsearch.who.int/Trial2.aspx?TrialID=EUCTR2011-002175-42-AT, 2011.

5. Nct, Pilot Study Comparing Treatment With Dexmedetomidine to Midazolam for Symptom Control in Advanced Cancer Patients. https://clinicaltrials.gov/show/NCT01687751, 2012.

6. Nct, Effect of Intravenous Dexmedetomidine on Analgesic Duration of Infraclavicular Block. https://clinicaltrials.gov/show/NCT01981369, 2013.

7. Nct, Dexmedetomidine and Delirium in Patients After Cardiac Surgery. https://clinicaltrials.gov/show/NCT02267538, 2014.

8. Su, X., et al., Prophylactic low-dose dexmedetomidine decreases the incidence of delirium in critically ill elderly patients after noncardiac surgery: A randomized controlled trial. Intensive Care Medicine, 2014. 40(1): p. S245.

9. Euctr, F.I., The Neural Mechanisms of Anesthesia and Human Consciousness (Part 6). https://trialsearch.who.int/Trial2.aspx?TrialID=EUCTR2015-004982-10-FI, 2015.

10. Chen, C., et al., Dexmedetomidine improves gastrointestinal motility after laparoscopic resection of colorectal cancer: A randomized clinical trial. Medicine (United States), 2016. 95(29).

11. Kamal, M., et al., Efficacy of dexmedetomidine as an adjuvant to ropivacaine in pediatric caudal epidural block. Saudi Journal of Anaesthesia, 2016. 10(4): p. 384-389.

12. Kanji, S., et al., Pharmacological interventions to improve sleep in hospitalised adults: a systematic review. BMJ Open, 2016. 6(7): p. e012108.

13. Loh, P.S., et al., Comparing the efficacy and safety between propofol and dexmedetomidine for sedation in claustrophobic adults undergoing magnetic resonance imaging (PADAM trial). Journal of clinical anesthesia, 2016. 34: p. 216Γאנ222.

14. Su, X., et al., Dexmedetomidine for prevention of delirium in elderly patients after non-cardiac surgery: a randomised, double-blind, placebo-controlled trial. The Lancet, 2016. 388(10054): p. 1893-1902.

15. Yu, J.M., et al., The Analgesic Effect of Ropivacaine Combined With Dexmedetomidine for Incision Infiltration After Laparoscopic Cholecystectomy. Surgical laparoscopy, endoscopy & percutaneous techniques, 2016. 26(6): p. 449Γאנ454.

16. Builes, A., et al., Effect of intravenous dexmedetomidine in the duration of infraclavicular block: Randomized, double-blind study. Canadian Journal of Anesthesia, 2017. 64(1): p. S161-S163.

17. Hollinger, A., et al., Comparison of propofol and dexmedetomidine infused overnight to treat hyperactive and mixed ICU delirium: a protocol for the Basel ProDex clinical trial. BMJ open, 2017. 7(7): p. e015783.

18. Nct, Dexmedetomidine Supplemented Analgesia and 2-Year Survival After Cancer Surgery. https://clinicaltrials.gov/show/NCT03012971, 2017.

19. Nct, Low-dose Dexmedetomidine in Mechanically Ventilated ICU Patients. https://clinicaltrials.gov/show/NCT03172897, 2017.

20. Nct, Dexmedetomidine and Long-term Outcomes in Elderly Patients After Cardiac Surgery. https://clinicaltrials.gov/show/NCT03289325, 2017.

21. Qin, M., et al., Dexmedetomidine in combination with sufentanil for postoperative analgesia after partial laryngectomy. BMC ANESTHESIOLOGY, 2017. 17.

22. Kwon, M., N. Patel, and V. Arunthari, Nocturnal dexmedetomidine for prevention of delirium in the ICU. Journal of Clinical Outcomes Management, 2018. 25(8): p. 354-356.

23. Nct, Low-dose Dexmedetomidine and Postoperative Delirium After Cardiac Surgery. https://clinicaltrials.gov/show/NCT03624595, 2018.

24. Nct, Dexmedetomidine Combined With Ropivacaine for Postoperative Continuous Femoral Nerve Block. https://clinicaltrials.gov/show/NCT03629483, 2018.

25. Nct, Dexmedetomidine Supplemented Intravenous Analgesia in Elderly After Orthopedic Surgery. https://clinicaltrials.gov/show/NCT03629262, 2018.

26. Nct, Propofol and Dexmedetomidine on Inflammation. https://clinicaltrials.gov/show/NCT03600727, 2018.

27. Nct, Effect of Dexmedetomidine on Postoperative Delirium Inflammasome Activation Inhibition. https://clinicaltrials.gov/show/NCT03588988, 2018.

28. Wang, B.J., et al., Impact of dexmedetomidine infusion during general anaesthesia on incidence of postoperative delirium in elderly patients after major non-cardiac surgery: study protocol of a randomised, double-blinded and placebo-controlled trial. BMJ open, 2018. 8(4): p. e019549.

29. Hong, B., et al., Prolonged analgesic duration of brachial plexus block by addition of dexamethasone to dexmedetomidine for sedation. Regional Anesthesia and Pain Medicine, 2019. 44(10): p. A271-A273.

30. Huyan, T., et al., Perioperative dexmedetomidine reduces delirium in elderly patients after lung cancer surgery. Psychiatria Danubina, 2019. 31(1): p. 95-101.

31. Nct, Dexmedetomidine Sedation in Orthopedic Surgery. https://clinicaltrials.gov/show/NCT04149626, 2019.

32. Sun, Y., et al., Impact of postoperative dexmedetomidine infusion on incidence of delirium in elderly patients undergoing major elective noncardiac surgery: a randomized clinical trial. DRUG DESIGN DEVELOPMENT AND THERAPY, 2019. 13: p. 2911-2922.

33. Wu, L., et al., Effect of ultrasound-guided peripheral nerve blocks of the abdominal wall on pain relief after laparoscopic cholecystectomy. Journal of Pain Research, 2019. 12: p. 1433-1439.

34. ChiCtr, Effect of intrEffect of intravertebral anesthesia assisted dexmedetomidine sedation on circadian rhythm in patients undergoing lower limb orthopedic surgery. http://www.who.int/trialsearch/Trial2.aspx?TrialID=ChiCTR2000028954, 2020.

35. Gandolfi, J.V., et al., The Effects of Melatonin Supplementation on Sleep Quality and Assessment of the Serum Melatonin in ICU Patients: A Randomized Controlled Trial. Critical Care Medicine, 2020. 48(12): p. E1286-E1293.

36. Liu, X., et al., Combination of post-fascia iliaca compartment block and dexmedetomidine in pain and inflammation control after total hip arthroplasty for elder patients: a randomized control study. Journal of orthopaedic surgery and research, 2020. 15(1): p. 42.

37. Mao, Y., et al., Perioperative Dexmedetomidine Fails to Improve Postoperative Analgesic Consumption and Postoperative Recovery in Patients Undergoing Lateral Thoracotomy for Thoracic Esophageal Cancer: A Randomized, Double-Blind, Placebo-Controlled Trial. PAIN RESEARCH & MANAGEMENT, 2020.

38. Shi, H., et al., Dexmedetomidine improves early postoperative neurocognitive disorder in elderly male patients undergoing thoracoscopic lobectomy. Experimental and Therapeutic Medicine, 2020. 20(4): p. 3867-3876.

39. Cheng, X.Q., et al., Anti-nociceptive effects of dexmedetomidine infusion plus modified intercostal nerve block during single-port thoracoscopic lobectomy: A double-blind, randomized controlled trial. Pain Physician, 2021. 24(5): p. E565-E572.

40. Euctr, D.K., The effects of combined use of dexamethasone (corticosteroid) and dexmedetomidine (sedative) on a nerve block in patients undergoing surgery of the bones in the foot and ankle. https://trialsearch.who.int/Trial2.aspx?TrialID=EUCTR2021-000429-28-DK, 2021.

41. Hong, B., et al., The effect of intravenous dexamethasone and dexmedetomidine on analgesia duration of supraclavicular brachial plexus block: A randomized, four-arm, triple-blinded, placebo-controlled trial. Journal of Personalized Medicine, 2021. 11(12).

42. Hong, H., et al., Impact of dexmedetomidine supplemented analgesia on delirium in patients recovering from orthopedic surgery: a randomized controlled trial. BMC anesthesiology, 2021. 21(1): p. 223.

43. Jin, X.B., et al., Effect of Different Modes of Administration of Dexmedetomidine Combined with Nerve Block on Postoperative Analgesia in Total Knee Arthroplasty. Pain and Therapy, 2021. 10(2): p. 1649-1662.

44. Lu, Y., et al., Effect of Intraoperative Dexmedetomidine on Recovery of Gastrointestinal Function After Abdominal Surgery in Older Adults: a Randomized Clinical Trial. JAMA network open, 2021. 4(10): p. e2128886.

45. Nct, Dexmedetomidine Supplemented Analgesia and Delirium After Hip Fracture Surgery. https://clinicaltrials.gov/show/NCT04955249, 2021.

46. Nct, Low-dose S-ketamine and Dexmedetomidine in Combination With Opioids for Postoperative Analgesia. https://clinicaltrials.gov/show/NCT04791059, 2021.

47. Nct, Comparison of Doses of Dexmedetomidine With Bupivacaine in Caudal Block for Duration of Analgesia in Paediatric Infraumblical Surgeries. https://clinicaltrials.gov/show/NCT04700228, 2021.

48. Niu, Z., et al., Effect of total intravenous anesthesia or inhalation anesthesia on postoperative quality of recovery in patients undergoing total laparoscopic hysterectomy: A randomized controlled trial. Journal of Clinical Anesthesia, 2021. 73.

49. Rekatsina, M., P. Theodosopoulou, and C. Staikou, Effects of Intravenous Dexmedetomidine Versus Lidocaine on Postoperative Pain, Analgesic Consumption and Functional Recovery After Abdominal Gynecological Surgery: A Randomized Placebo-controlled Double Blind Study. PAIN PHYSICIAN, 2021. 24(7): p. E997-E1006.

50. Singh, A., et al., Analgesic Efficacy and Safety of Dexmedetomidine as an Adjuvant to Caudal Levobupivacaine for Infraumbilical Surgeries in Children. Anestezi Dergisi, 2021. 29(4): p. 263-269.

51. Tan, J., et al., [Effect of CT localization of upper airway obstruction site after inducing sleep on the value of obstructive sleep apnea hypopnea syndrome and the effect of surgery]. Lin Chung Er Bi Yan Hou Tou Jing Wai Ke Za Zhi, 2021. 35(8): p. 683-688.

52. Wang, D., et al., Effect of dexmedetomidine on postoperative delirium in patients undergoing brain tumour resections: Study protocol of a randomised controlled trial. BMJ Open, 2021. 11(11).

53. Wang, J., et al., Clinical Effect of Different Drugs and Infusion Techniques for Patient-controlled Analgesia After Spinal Tumor Surgery: A Prospective, Randomized, Controlled Clinical Trial. CLINICAL THERAPEUTICS, 2021. 43(6): p. 1020-1028.

54. Zhang, J.F., et al., Multimodal sleep, an innovation for treating chronic insomnia: case report and literature review. J Clin Sleep Med, 2021. 17(8): p. 1737-1742.

55. Atri, A., M. Devi, and N. Gupta, Efficacy of Dexmedetomidine as an Adjuvant to Levobupivacaine in Caudal Anaesthesia in Paediatric Patients. International Journal of Pharmaceutical and Clinical Research, 2022. 14(7): p. 254-261.

56. Fan, Q., et al., Transcutaneous Electrical Acupoint Stimulation Combined With Auricular Acupressure Reduces Postoperative Delirium Among Elderly Patients Following Major Abdominal Surgery: A Randomized Clinical Trial. Frontiers in Medicine, 2022. 9.

57. Fondeur, J., et al., Dexmedetomidine in Prevention of Postoperative Delirium: A Systematic Review. Cureus, 2022. 14(6): p. e25639.

58. Huang, X., et al., Ultrasound-guided erector spinae plane block improves analgesia after laparoscopic hepatectomy: a randomised controlled trial. British Journal of Anaesthesia, 2022. 129(3): p. 445-453.

59. Lee, E.Y. and M.E. Wilcox, Sleep in the intensive care unit. Curr Opin Pulm Med, 2022. 28(6): p. 515-521.

60. Liu, T., et al., Effect of Perioperative Dexmedetomidine Infusion on Postoperative Delirium in Elderly Patients Undergoing Oral and Maxillofacial Surgery: A Randomized Controlled Clinical Trial. International Journal of General Medicine, 2022. 15: p. 6105-6113.

61. Nct, The Effects of Subanesthetic S-ketamine on Postoperative Delirium and Cognitive Function in the Elderly Undergoing Non-cardiac Thoracic Surgery. https://clinicaltrials.gov/show/NCT05242692, 2022.

62. Wang, W., et al., Dexmedetomidine infusion for emergence coughing prevention in patients undergoing an endovascular interventional procedure: A randomized dose-finding trial. EUROPEAN JOURNAL OF PHARMACEUTICAL SCIENCES, 2022. 177.

63. Wei, W., et al., Effects of subanaesthetic S-ketamine on postoperative delirium and cognitive function in elderly patients undergoing non-cardiac thoracic surgery: a protocol for a randomised, double-blinded, placebo-controlled and positive-controlled, non-inferiority trial (SKED trial). BMJ Open, 2022. 12(8).

64. Xu, S., et al., Effect of co-administration of intravenous lidocaine and dexmedetomidine on the recovery from laparoscopic hysterectomy: a randomized controlled trial. Minerva anestesiologica, 2022.

- **Articles excluded for the following reasons after full-text screening (n = 47):**

**Articles that were study protocols (n = 21)**

1. Nct, A Pilot Study of Effect of Dexmedetomidine on Sleep and Inflammation in Critically Ill Patients. https://clinicaltrials.gov/show/NCT00405847, 2006.
2. Nct, Postoperative Sleep Quality in Patients Undergoing Thoracic Surgery With Different Types of Anesthesia Management. https://clinicaltrials.gov/show/NCT01725607, 2012.
3. Nct, Does Nightly Dexmedetomidine Improve Sleep and Reduce Delirium in ICU Patients? https://clinicaltrials.gov/show/NCT01791296, 2013.
4. Nct, Postoperative Sleep Quality of Patients Sedation With i.v. Dexmedetomidine or Midazolam Undergoing TURP. https://clinicaltrials.gov/show/NCT02142595, 2014.
5. Chi, C.I., Effects of oxycodone hydrochloride combined dexmedetomidine for postoperative analgesia on sleep quality in patients after major gastrointestinal surgery. https://trialsearch.who.int/Trial2.aspx?TrialID=ChiCTR-INR-17010315, 2017.
6. Euctr, D.K., A comparison of dexmedetomidine vs placebo effect on sleep-quality in mechanical ventilated critical ill patients. https://trialsearch.who.int/Trial2.aspx?TrialID=EUCTR2017-001612-11-DK, 2017.
7. Nct, Impact of Dexmedetomidine on Sleep Quality. https://clinicaltrials.gov/show/NCT03117790, 2017.
8. Nct, Impacts of Low-Dose Dexmedetomidine on Sleep Quality in Mechanically Ventilated ICU Patients. https://clinicaltrials.gov/show/NCT03335527, 2017.
9. Nct, Investigation of Sleep in the Intensive Care Unit. https://clinicaltrials.gov/show/NCT03355053, 2017.
10. Nct, the Influence of Sleep Improvement Through Different Methods on Patients in ICU. https://clinicaltrials.gov/show/NCT03480789, 2018.
11. Nct, Dexmedetomidine and Outcomes of Elderly Admitted to ICU After Surgery. https://clinicaltrials.gov/show/NCT04204798, 2019.
12. Nct, Dexmedetomidine Supplemented Analgesia in Patients at High-risk of Obstructive Sleep Apnea. https://clinicaltrials.gov/show/NCT04608331, 2020.
13. Nct, DexmedetOmidine Complement Treats Chronic insOmnia and Improves Circadian Rhythm (DOCTOR). https://clinicaltrials.gov/show/NCT04635098, 2020.
14. Nct, Co-administration of Dexmedetomidine in Carotid Endarterectomy (CEA). https://clinicaltrials.gov/show/NCT04662177, 2020
15. Nct, Dexmedetomidine Adjuvant Treatment for Depressed Patients Undergoing ECT. https://clinicaltrials.gov/show/NCT04661475, 2020.
16. Nct, Dexmedetomidine Cycling and Sleep in the Pediatric ICU. https://clinicaltrials.gov/show/NCT05003102, 2021.
17. Nct, The Neuroprotective Effect of Dexmedetomidine Preserving Brain Functional Connectivity in Elderly Patients. https://clinicaltrials.gov/show/NCT04973124, 2021.
18. Irct20161127031131N, Comparison of the effect of dexmedetomidine and propofol on how patients sleep after cardiac surgery. https://trialsearch.who.int/Trial2.aspx?TrialID=IRCT20161127031131N3, 2022.
19. Nct, Effect of Propofol on Postoperative Sleep Quality in Elderly Patients With Sleep Disorders. https://clinicaltrials.gov/show/NCT05325762, 2022.
20. Nct, Nocturnal Low-dose Dexmedetomidine Infusion and Perioperative Sleep Quality. https://clinicaltrials.gov/show/NCT05246007, 2022.
21. Oxlund, J., et al., Dexmedetomidine and sleep quality in mechanically ventilated critically ill patients: study protocol for a randomised placebo-controlled trial. BMJ Open, 2022. 12(3): p. e050282.

**Articles that had no PSG data (n = 15)**

1. Nct, Dexmedetomidine as an Adjuvant for Sub-Tenon's Anesthesia. https://clinicaltrials.gov/show/NCT02327156, 2014.
2. Ghali, A.M., A.M. Shabana, and A.M. El Btarny, The Effect of Low-Dose Dexmedetomidine as an Adjuvant to Levobupivacaine in Patients Undergoing Vitreoretinal Surgery Under Sub-Tenon's Block Anesthesia. ANESTHESIA AND ANALGESIA, 2015. 121(5): p. 1378-1382.
3. Tan, W.F., et al., Changes in postoperative night bispectral index of patients undergoing thoracic surgery with different types of anaesthesia management: a randomized controlled trial. Clinical and experimental pharmacology & physiology, 2016. 43(3): p. 304Γאנ311.
4. Tan, W.F., et al., Changes in First Postoperative Night Bispectral Index After Daytime Sedation Induced by Dexmedetomidine or Midazolam Under Regional Anesthesia: a Randomized Controlled Trial. Regional anesthesia and pain medicine, 2016. 41(3): p. 380Γאנ386.
5. Lu, W., et al., Effects of dexmedetomidine on sleep quality of patients after surgery without mechanical ventilation in ICU. Medicine (Baltimore), 2017. 96(23): p. e7081.
6. Skrobik, Y., et al., Impact of nocturnal dexmedetomidine on delirium incidence and sleep quality in critically ill adults: A randomized, double-blind, placebo-controlled trial. American Journal of Respiratory and Critical Care Medicine, 2017. 195.
7. Li, H.J., et al., Dexmedetomidine in combination with morphine improves postoperative analgesia and sleep quality in elderly patients after open abdominal surgery: A pilot randomized control trial. PLoS One, 2018. 13(8): p. e0202008.
8. Skrobik, Y., et al., Low-Dose Nocturnal Dexmedetomidine Prevents ICU Delirium A Randomized, Placebo-controlled Trial. AMERICAN JOURNAL OF RESPIRATORY AND CRITICAL CARE MEDICINE, 2018. 197(9): p. 1147-1156.
9. Wang, X., et al., Dexmedetomidine combined with ropivacaine for continuous femoral nerve block improved postoperative sleep quality in elderly patients after total knee arthroplasty. National Medical Journal of China, 2018. 98(10): p. 728-732.
10. Yu, H.Y., et al., Dexmedetomidine Alleviates Postpartum Depressive Symptoms following Cesarean Section in Chinese Women: A Randomized Placebo-Controlled Study. Pharmacotherapy, 2019. 39(10): p. 994-1004.
11. An, J.X., et al., Feasibility of Patient-Controlled Sleep with Dexmedetomidine in Treating Chronic Intractable Insomnia. Nat Sci Sleep, 2020. 12: p. 1033-1042.
12. Duprey, M.S., J.W. Devlin, and Y. Skrobik, Is there an association between subjective sleep quality and daily delirium occurrence in critically ill adults? A post hoc analysis of a randomised controlled trial. BMJ OPEN RESPIRATORY RESEARCH, 2020. 7(1).
13. Wang, Y., et al., Dexmedetomidine-soaked nasal packing can reduce pain and improve sleep quality after nasal endoscopic surgery: a double-blind, randomized, controlled clinical trial. SLEEP AND BREATHING, 2021. 25(4): p. 2045-2052.
14. Shi, J., et al., Effects of Dexmedetomidine Combined with Intravenous Anesthesia on Oxidative Stress Index, Postoperative Sleep Quality, and Brain Function in HICH Patients. J Healthc Eng, 2022. 2022: p. 5463986.
15. Sui, X., et al., The effects of dexmedetomidine for patient-controlled analgesia on postoperative sleep quality and gastrointestinal motility function after surgery: A prospective, randomized, double-blind, and controlled trial. Frontiers in Pharmacology, 2022. 13.

**Articles that were not for surgical patients (n = 7)**

1. Oto, J., et al., Sleep quality of mechanically ventilated patients sedated with dexmedetomidine. INTENSIVE CARE MEDICINE, 2012. 38(12): p. 1982-1989.
2. Li, X., et al., Efficacy of dexmedetomidine with cognitive behavioral therapy for treating chronic insomnia related to conditioned arousal: a randomized controlled trial. Sleep and biological rhythms, 2016. 14(1): p. 75Γאנ85.
3. Akeju, O., et al., Dexmedetomidine promotes biomimetic non-rapid eye movement stage 3 sleep in humans: A pilot study. Clinical Neurophysiology, 2018. 129(1): p. 69-78.
4. Ueno, Y. and J. Oto, Effects of nocturnal dexmedetomidine on sleep quality in critically ill patients equipped with high flow nasal cannula. Intensive Care Medicine Experimental, 2018. 6.
5. Chamadia, S., et al., Oral Dexmedetomidine Promotes Non-rapid Eye Movement Stage 2 Sleep in Humans. Anesthesiology, 2020. 133(6): p. 1234Γאנ1243.
6. Oxlund, J., et al., Sleep quality and quantity determined by polysomnography in mechanically ventilated critically ill patients randomized to dexmedetomidine or placebo. Acta Anaesthesiologica Scandinavica, 2022.
7. Ueno, Y., et al., The quality and quantity of sleep on dexmedetomidine during high-flow nasal cannula oxygen therapy in critically ill patients. The journal of medical investigation : JMI, 2022. 69(3.4): p. 266-272.

**Articles that were reviews (n = 1)**

1. Huang, X., et al., Effect of Dexmedetomidine on Postoperative Sleep Quality: A Systematic Review. Drug Des Devel Ther, 2021. 15: p. 2161-2170.

**Articles that were not adult study (n = 1)**

1. Zhang, X., et al., Dexmedetomidine Improves Non-rapid Eye Movement Stage 2 Sleep in Children in the Intensive Care Unit on the First Night After Laparoscopic Surgery. Front Pediatr, 2022. 10: p. 871809.

**Articles that were not RCT (n = 1)**

1. Cai, J., et al., Effect of Intraoperative Dexmedetomidine Dose on Postoperative First Night Sleep Quality in Elderly Surgery Patients: A Retrospective Study With Propensity Score-Matched Analysis. Front Med (Lausanne), 2020. 7: p. 528.

**Articles that had no control group (n = 1)**

1. Song, B., et al., The Effect Of Intraoperative Use Of Dexmedetomidine During The Daytime Operation Vs The Nighttime Operation On Postoperative Sleep Quality And Pain Under General Anesthesia. Nat Sci Sleep, 2019. 11: p. 207-215.

- **Five studies were included in our meta-analysis**

1. Chen Z, Tang R, Zhang R, Jiang Y, Liu Y: Effects of dexmedetomidine administered for postoperative analgesia on sleep quality in patients undergoing abdominal hysterectomy. J CLIN ANESTH 2017, 36:118-122.
2. Wu Y, Miao Y, Chen X, Wan X: A randomized placebo-controlled double-blind study of dexmedetomidine on postoperative sleep quality in patients with endoscopic sinus surgery. BMC ANESTHESIOL 2022, 22(1):172.
3. Jiang Z, Zhou G, Song Q, Bao C, Wang H, Chen Z: Effect of Intravenous Oxycodone in Combination With Different Doses of Dexmedetomdine on Sleep Quality and Visceral Pain in Patients After Abdominal Surgery: A Randomized Study. CLIN J PAIN 2018, 34(12):1126-1132.
4. Sun YM, Zhu SN, Zhang C, Li SL, Wang DX: Effect of low-dose dexmedetomidine on sleep quality in postoperative patients with mechanical ventilation in the intensive care unit: A pilot randomized trial. FRONT MED-LAUSANNE 2022, 9:931084.
5. Wu XH, Cui F, Zhang C, Meng ZT, Wang DX, Ma J, Wang GF, Zhu SN, Ma D: Low-dose Dexmedetomidine Improves Sleep Quality Pattern in Elderly Patients after Noncardiac Surgery in the Intensive Care Unit: A Pilot Randomized Controlled Trial. ANESTHESIOLOGY 2016, 125(5):979-991.
